# Systematic profiling of mRNA and miRNA expression in the pancreatic islets of spontaneously diabetic Goto-Kakizaki rats

**DOI:** 10.3892/mmr.2014.2723

**Published:** 2014-10-21

**Authors:** LING-QIN ZENG, SU-BI WEI, YI-MIN SUN, WEN-YAN QIN, JING CHENG, KEITH MITCHELSON, LAN XIE

**Affiliations:** 1 Medical Systems Biology Research Center, Tsinghua University School of Medicine, Beijing 100084, P.R. China; 2 Department of Biomedical Engineering, Tsinghua University School of Medicine, Beijing 100084, P.R. China; 3 The State Key Laboratory of Biomembrane and Membrane Biotechnology, Tsinghua University, Beijing 100084, P.R. China; 4 National Engineering Research Center for Beijing Biochip Technology, Beijing 102206, P.R. China

**Keywords:** type 2 diabetes, gene expression array, microRNA microarray, microRNA-mRNA regulatory correlation

## Abstract

Type 2 diabetes (T2DM) is a complex multifactorial metabolic disorder that affects >100 million individuals worldwide, yet the mechanisms involved in the development and progression of the disease have not yet been fully elucidated. The present study examined the mRNA and micro (mi)RNA expression profiles by microarray analysis in the pancreas islets of spontaneously diabetic Goto-Kakizaki rats with the aim to identify regulatory mechanisms underlying the pathogenesis of T2DM. A total of 9 upregulated and 10 downregulated miRNAs were identified, including miR-150, miR-497, miR-344-3p and let-7f, which were independently validated by quantitative polymerase chain reaction assays. In addition, differential expression of 670 genes was detected by mRNA microarray analysis, including 370 upregulated and 247 downregulated genes. The differentially expressed genes were statistically associated with major cellular pathways, including the immune response pathway and the extracellular matrix (ECM)-receptor interaction pathway. Finally, a reverse regulatory association of differentially expressed miRNAs and their predicted target genes was constructed, supported by analysis of their mRNA and miRNA expression profiles. A number of key pairs of miRNA-mRNA was proposed to have significant roles in the pathogenesis of T2DM rats based on bioinformatics analysis, one example being the let-7f/collagen, type II, alpha 1 pair that may regulate ECM-receptor interactions.

## Introduction

Diabetes mellitus (or diabetes) is a chronic disease that seriously affects human health. The World Health Organization estimated ~285 million diabetes sufferers worldwide in 2010, and this number is expected to increase to 360 million by 2030 (1). In total, ~90% of the cases are Type 2 diabetes (T2DM), which has a more complex pathogenesis than Type 1. T2DM is a multi-factorial disease characterized by peripheral insulin resistance (2), occasionally combined with an absolute lack of insulin.

Several large genetic association studies and genome-wide association studies have revealed significant genes consistently associated with susceptibility to T2DM, including cyclin-dependent kinase inhibitor 2A/2B, CDK5 regulatory subunit associated protein 1-like 1, insulin-like growth factor 2 mRNA binding protein 2 and others (3). Although these findings have facilitated the understanding of the genetic causes of T2DM, this is not sufficient to elucidate the progress and development of the disease, since the relative expression of genes is also altered in a tissue- and time-specific manner as the disease progresses.

Gene expression microarrays are a high-throughput technology, which can be used for systematic studies of complex molecular processes, including tissue development and differentiation and for diseases, including cancer and diabetes. Gene expression microarrays can give a genome-wide view of the way genes are regulated at the transcriptional level in different tissues at different time-points. Over the last two decades microRNAs (miRNAs) have been found to act as key regulators of the expression of multiple genes at the post-transcriptional level (4), and it is now understood that ~30% of all the coding genes in the human genome are regulated by miRNAs (5), including genes involved in cell proliferation and differentiation, apoptosis and metabolism (6). Additionally, miRNAs also have roles in pathogenic processes, including cancer (7,8) and inflammatory diseases (9).

Numerous gene expression studies have been undertaken on T2DM, either on the pancreatic islets (10) or peripheral organs, including muscle (11), kidney (12), liver (13) and adipose tissues (14). However, the simultaneous examination of mRNA and miRNA gene expression and analysis of their association in the same T2DM model tissue has not been previously elucidated. In the present study, the mRNA and miRNA expression profiles of pancreatic tissue from T2DM Goto-Kakizaki (GK) rats were examined, and miRNA-mRNA regulatory networks were constructed based on statistical analysis of the data, which are noteworthy of further functional analysis.

## Materials and methods

### Animals

GK rats were purchased from the Shanghai SiLaike Laboratory Animal Company (Shanghai, China) and Wistar rats were from the Beijing Weitonglihua Laboratory Animal Center (Beijing, China). All the rats were housed in the Animal House of Tsinghua University (Beijing, China) and used in compliance with the National Insurance of Health Guidance for the Care and Use of Laboratory Animals. The 13–14 week-old male GK and Wistar rats (300–400 g) were fed a normal diet. The present study was approved by the Ethics Committee of Tsinghua University School of Medicine (Beijing, China).

### Islet preparation and RNA extraction

Pancreas islets were isolated from the pancreas of the male GK and Wistar rats using a collagenase digestion technique. In brief, the pancreases were distended via intraductal injection of 3 ml 0.5 mg/ml collagenase (type V, C-9263, Invitrogen Life Technologies, Carlsbad, CA, USA) into the duodenal nipple following occlusion of the common bile duct. The pancreases were then surgically removed and further digested in 2 ml 0.5 mg/ml collagenase for 30 min at 37°C. Islet digests were then vigorously agitated and quenched by the addition of ice-cold Hank’s balanced salt solution (HBSS) supplemented with 12.5% fetal bovine serum (FBS; Gibco Laboratories, Grand Island, NY, USA). The suspension was washed twice to remove collagenase (in HBSS, 1,500 xg, 10 sec, 4°C) in order to separate the islets from the surrounding undigested tissue.

Pancreatic islet cell resuspensions, with 70–80% purity of islet cells, were cultured in RPMI-1640 medium (Gibco Laboratories) with 15% FBS (v/v), 100 μg/ml penicillin, and then incubated at 37°C under humidified conditions with 5% CO_2_. RNA extraction for the tissue samples was undertaken with TRIzol (Invitrogen Life Technologies) following the manufacturer’s instructions.

### miRNA microarrays

Comprehensive miRNA profiling was performed using the GeneChip^®^ miRNA 2.0 Array (Affymetrix, Santa Clara, CA, USA) according to the manufacturer’s instructions. Briefly, RNA from three GK or Wistar rat islets was mixed into a pool, respectively, and 1 μg total RNA was labeled with the Biotin FlashTag Biotin Labeling Kit (Affymetrix). The labeling reaction was hybridized on the miRNA array in an Affymetrix Hybridization Oven 640 (Affymetrix). The arrays were stained on the Fluidics Station 450 and then scanned on a GeneChip^®^ Scanner 3000 (both Affymetrix).

### Quantitative polymerase chain reaction (qPCR)

qPCR analysis was performed in order to verify the expression results obtained with the Affymetrix miRNA expression platform (Affymetrix). Stem-loop reverse transcription (RT)-PCR assays were performed according to the method by Chen *et al* (15) using a SuperGreen quantitative PCR kit (CapitalBio Corp., Beijing, China) in a LightCycler (Roche Diagnostics, Basel Switzerland). The relative expression was calculated using the following formula: Q=2^−ΔΔCT^, where ΔΔCT=ΔCT_sample_ - ΔCT_control_; ΔCT=average CT _test miRNA_ - averageCT _internal control_, and CT stands for cycle threshold. Let-7c was used as an internal standard. The stem-loop RT primer and PCR primers for each miRNA are available upon request. Statistical significance was determined by a student’s t-test, using SPSS software version 20.0 (SPSS, Inc, Chicago, IL, USA). P<0.05 was considered to indicate a statistically significant difference.

### Gene expression microarrays

The 27K Rat Genome Array chips (SmartArray^TM^, CapitalBio Corp), were used to profile gene expression in islets from GK/Wistar rats according to the manufacturer’s instructions. Briefly, RNA from three GK or Wistar rat islets was mixed into a pool, respectively and the total RNA samples (100 ng) were reversely transcribed using Moloney Murine leukemia virus (Takara Chemicals, Shiga, Japan) and amplified by an *in vitro* transcription based method. The Cyanine 3 (Cy3) or Cyanine 5 (Cy5)-labeled cDNAs were hybridized to the CapitalBio 27K rat genome array for 16 h at 42°C. The arrays were scanned with a LuxScan^TM^ 10K-A confocal scanner, and the data were extracted with GenePix pro4.0 software (both CapitalBio).

### Gene Ontology analysis and pathway identification

The web-based Molecule Annotation System [(MAS): http://bioinfo.capitalbio.com/mas] was used to identify the possible functions of the differentially-expressed genes. The MAS is a data-mining and function-annotation tool using all the known genes in the human genome as a background. The P-value of the pathway was calculated using a hyper-geometric distribution. The Q-value is a measure in terms of the false discovery rate (FDR). The pathways with Q-value<0.05 were considered statistically significantly enriched in the input set of host genes.

### Confident prediction of microRNA targets combined with gene expression profiles

#### Bioinformatics prediction of miRNA targets

Two different databases were used for miRNA target predictions, MiRanda (www.microrna.org/microrna/getDownloads.do) and Targetscan (www.targetscan.org/mmu_60/), and only those targets predicted by both databases were selected.

#### Combining predicted targets with gene expression profiles

Typically, miRNAs downregulate target gene expression, thus it was assumed that the expression of a given miRNA would be reversely correlated with the mRNA expression of its targets. Based on bioinformatics prediction, only microRNA-target gene pairs with such opposing expression patterns were selected.

#### Functional enrichment analysis of miRNA target genes

The parameter θ was used to reflect the miRNA-target regulation strength. θ is defined as (|FCm-1/FCg|), where FCm represents the fold change in miRNA expression and FCg represents the fold change in target gene expression. A stronger miRNA-target gene regulatory correlation (*i.e.* a higher θ value) theoretically indicates a more significant regulatory correlation between miRNA and the target gene.

## Results

### miRNA microarray analysis and qPCR confirmation of GK rats

**Figure 1:**
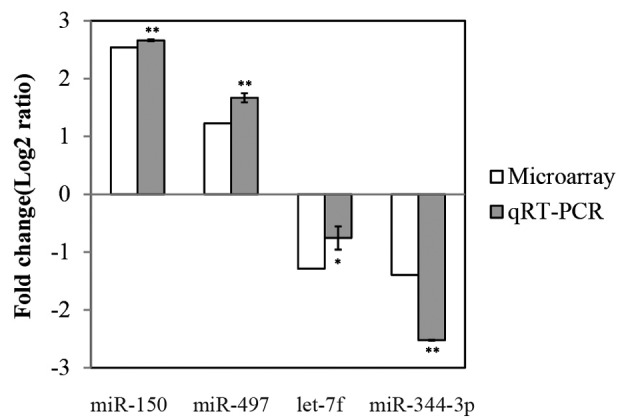
qRT-PCR validation of representative differentially-expressed miRNAs identified from the microarray. Bars represent fold changes comparing expression between GK and Wistar rats. A positive/negative fold change represents upregulation/downregulation, respectively, of expression in GK rats. Error bars represent the standard deviation. ^*^P<0.05; ^**^P<0.01. qRT-PCR, quantitative real-time polymerase chain reaction; GK, Goto-Kakizaki; RT, realtime; miRNA, microRNA.

miRNA expression analysis was performed by microarray of islets from diabetic GK rats and normal Wistar rats and it was identified that a total of 19 miRNAs were >2-fold upregulated or downregulated in GK rats as compared with the controls (Table I). Among the miRNAs, miR-214, miR-199a-5p, miR-150, miR-199a-3p, miR-351, miR-145, miR-764, miR-497 and miR-92b were upregulated, whilst miR-7a, miR-325-5p, miR-485, miR-708, miR-344-3p, let-7f, miR-26b, miR-129, miR-29c and let-7a were downregulated. Stem-loop RT-PCR was used to confirm the expression levels of four selected miRNAs, in which miR-150 and miR-497 demonstrated upregulation, whereas miR-344-3p and let-7f showed downregulation, confirming the changes detected by the microarray (Fig. 1; P<0.05).

### Gene expression profiling of GK rats

**Figure 2:**
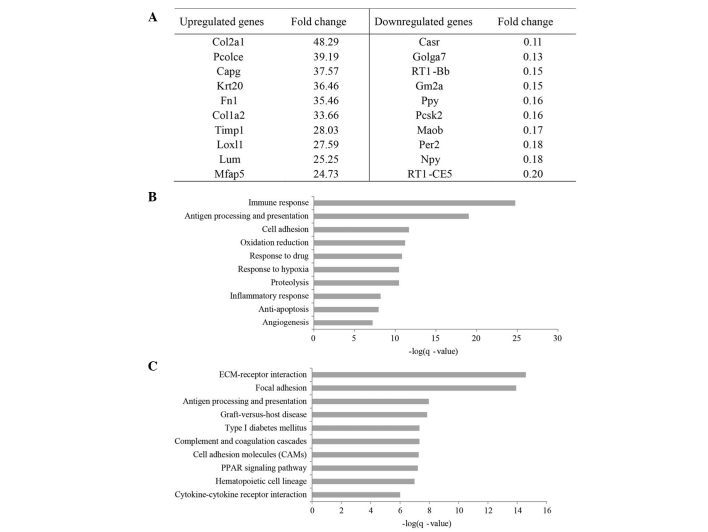
Functional analysis of the gene microarray results for GK rats. (A) Top ten differentially downregulated and -upregulated genes in GK rats compared to Wistar rats. (B) Top ten significantly enriched GO items (Biological process) for differentially-expressed genes between GK and Wistar rats. (C) Top ten significantly-enriched pathways (Kyoto Encyclopedia of Genes and Genomes) for differentially expressed genes between GK and Wistar rats. GK, Goto-Kakizaki; GO, Gene Ontology; *Col2a1*, collagen, type II, alpha 1; *Pcolce*, procollagen C-endopeptidase enhancer; *Capg*, macrophage-capping protein; Krt, keratin; Fn, fibronectin; *Colla2*, collagen, type I, alpha 2; *TIMP1*, tissue inhibitor of metalloproteinase-1; *Loxl1*, lysyl oxidase-like 1; *Lum*, lumican; *Mfap5*, microfibrillar associated protein 5; *Casr*, calcium-sensing receptor; *Golga7*, golgin A7; *Gm2a*, GM2 ganglioside activator; *Ppy*, pancreatic polypeptide; *Pcsk2*, proprotein convertase subtilisin/kexin type 2; *Maob*, monoamine oxidase B; *Per2*, period circadian clock 2; *Npy*, neuropeptide Y; ECM, extracellular matrix.

CaptitalBio 27K Rat Genome Array chips were used to evaluate the expression of target genes for differentially expressed miRNAs in GK rats. A total of 670 genes were differentially expressed (fold change >2.0) in GK rats compared to Wistar rats. Among them, 370 genes were found upregulated, while 247 were downregulated. The top ten differentially upregulated or downregulated genes are listed in Fig. 2A.

GO analysis (using the MAS bioinformatics tool) of the 670 differentially expressed genes revealed a number of statistically representative enriched GO items for biological processes, which are shown in Fig. 2B. The expression of genes with roles in immune responses, cell adhesion, oxidation/reduction and response to hypoxia were each significantly altered in GK rats.

MAS provided analysis of the canonical pathways in which the differentially expressed genes are involved. At an FDR of 1%, 27 pathways that were statistically significantly enriched in the identified genes were found, compared with the whole genome expression as the background. Of note, the identified pathways included extracellular matrix (ECM)-receptor interaction, focal adhesion, type I diabetes mellitus and peroxisome proliferator-activated receptor signaling pathways (Fig. 2C).

### Construction of the miRNA-mRNA regulatory networks

Typically, miRNAs regulate gene expression either through mRNA degradation or translational repression, depending on the degree of complementarity between miRNAs and their target gene sequences (16). In the present study, it was assumed that the expression of a given miRNA was inversely correlated with the mRNA expression of its putative targets. This bioinformatics prediction was combined with the miRNA/mRNA expression data of the present study to generate miRNA-mRNA regulatory networks.

Based on each of the putative miRNA-mRNA regulatory correlation, it was found that several miRNAs regulated more than one target gene, and that numerous genes were regulated by more than one miRNA. Among all the differentially expressed miRNAs, miR-485 had the most regulatory targets (32 target genes) and among the differentially expressed mRNAs, phosphoprotein enriched in astrocytes 15 (*Pea15a*), guanylate cyclase 1, soluble, alpha 3 (*Gucy1a3*), PDZK1 interacting protein 1 (*Pdzk1ip1*), connective tissue growth factor (*Ctgf*), inositol 1,4,5-triphosphate 3-kinase C (*Itpkc*) and heat shock protein 5 (*Hspa5*) had most regulatory miRNAs (each had 4 potential controlling miRNAs). Those miRNAs targeting multiple genes and those genes targeted by multiple miRNAs may be the nodal points of the whole regulatory network and may have more significant functions. The top 10 nodal miRNAs and mRNAs that had the most regulatory correlations are listed in Table II.

The significant levels of all pair-wise correlations between differentially-expressed miRNAs and mRNAs were evaluated through the parameter θ. In general, a stronger miRNA-mRNA regulatory correlation (*i.e.* a higher θ value) theoretically indicates more significant functions. The top 10 strongest miRNA-mRNA regulatory correlations are listed in Table III. Based on the results of the present study, the pair of miRNA-mRNA, let-7f/collagenase type II, alpha 1 (*Col2a1*) had the strongest regulatory correlation and could have significant roles in T2DM.

### Analysis of individual miRNA-mRNA regulatory correlation

Functional annotation analysis for the target genes of individual differentially expressed miRNA was undertaken, and 14 out of 19 differentially expressed miRNAs were identified to demonstrate at least one significantly enriched item of either GO or Pathway analysis for its target genes. These miRNAs include miR-214, miR-199a-5p, miR-150, miR-351, miR-145, miR-92b, miR-7a, miR-485, miR-708, let-7f, miR-26b, miR-129, miR-29c and let-7a.

**Figure 3:**
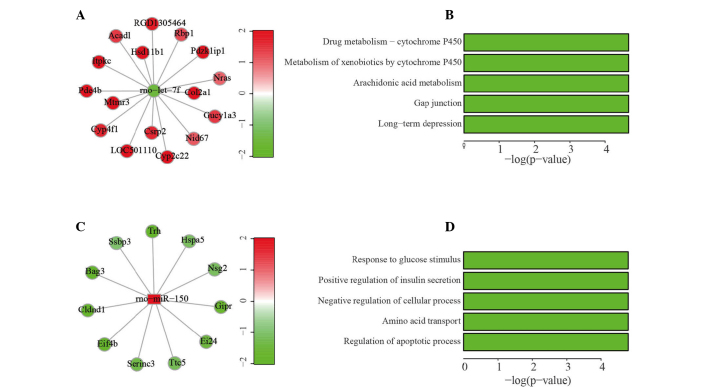
Construction of the representative miRNA-mRNA regulatory networks. (A) Regulatory network of let-7f. Green represents downregulation of the expression levels while red represents upregulation. (B) Pathway analysis (KEGG) of 16 target genes of let-7f. (C) Regulatory network of miR-150. (D) GO analysis of 11 target genes of miR-150. miRNA, microRNA; GO, Gene Ontology; *Rbp*, retinol binding protein; *Pdzk1ip1*, PDZK1 interacting protein 1; *Col2a1*, collagen, type II, alpha 1; *Gucy1a3*, guanylate cyclase 1; Nid, nidogen; Cyp, cytochrome P; *Csrp*, cysteine and glycine-rich protein; *Pde4B*, cyclic adenosine monophosphate-specific 3′,5′-cyclic phosphodiesterase 4B; *Mtmr3*, myotubularin-related protein 3; *Itpkc*, inositol-trisphosphate 3-kinase C; *Acadl*, acyl-CoA dehydrogenase, long chain; *Hsd11b1*, 11β-hydroxysteroid dehydrogenase type 1; *Hspa5*, heat shock protein 5; *Nsg2*, neuron-specific gene family member 2; *Gipr*, gastric inhibitory polypeptide receptor; *Ei24*, etoposide-induced protein 24; *Ttc5*, tetratricopeptide repeat domain 5; *Serinc3*, serine incorporator 3; *Eif4b*, eukaryotic translation initiation factor 4B; *Cldnd1*, claudin domain containing 1; *Bag3*, B-cell lymphoma 2-associated athanogene 3; *Ssbp3*, single-stranded DNA-binding protein 3; *Trh*, thyrotropin-releasing hormone.

For example, let-7f was found downregulated in GK rats by both the microarray and qPCR test and had a total of 16 target genes whose expression levels were elevated in GK rats (Fig. 3A). Pathway analysis of these 16 target genes indicated that numerous genes were involved in the drug metabolism, amongst others (Fig. 3B). By contrast, miR-150 was one of the upregulated miRNAs in GK rats and had 11 target genes downregulated in the GK rats (Fig. 3C). A GO analysis of these targets indicated that biological processes, including response to glucose stimulus, positive regulation of insulin secretion and others were overrepresented (Fig. 3D). The complete list of all the target genes of each of the 14 miRNAs and the complete list of significantly enriched items for target genes of each of the 14 miRNAs is available upon request.

## Discussion

The spontaneously diabetic GK rat is frequently used as a model for human T2DM. Molecular and functional changes of islet β-cells underlie the mechanism of how T2DM progresses, particularly in the very early stage (17). In several other studies, the combination of mRNA and miRNA profiling has also been used to characterize diseases and to provide substantial information with regard to regulatory correlations in these diseases, including in cancer (18,19), neurodegenerative disease (20,21) and infection (22). In the present study, the first combined analysis of the expression changes for both miRNAs and mRNAs in T2DM GK rats compared with normal Wistar rats were reported. Furthermore, miRNA-mRNA expression profiles were integrated through bioinformatics analysis and miRNA-mRNA regulatory networks that may have significant functions in T2DM were revealed.

Through the miRNA microarray, a total of 19 miRNAs were observed whose expression levels were significantly changed in GK rats compared to Wistar rats. Among the 19 miRNAs, four miRNAs have already been reported to be correlated to diabetes in previous studies, including miR-29c, let-7a, let-7f and miR-7 (23–25). He *et al* (23) reported an elevated expression of the miR-29 gene family in skeletal muscle, liver and adipose tissues of diabetic GK rats. Overexpression of miR-29a/b/c causes insulin resistance, similar to that of incubation with high glucose and insulin (23). Frost and Olson (24) demonstrated that both global and pancreas-specific overexpression of let-7 in mice resulted in impaired glucose tolerance and reduced glucose-induced pancreatic insulin secretion. Inhibition of the let-7 family prevents impaired glucose tolerance in mice with diet-induced obesity, partially by improving insulin sensitivity in the liver and muscle (24). Nieto *et al* (25) demonstrated that knockdown of miR-7 during early embryonic life resulted in an overall downregulation of insulin production, and decreased β-cell numbers and glucose intolerance in the postnatal period. Another two miRNAs, miR-199a-3p and miR-129, which were found differentially expressed in the present study, were also reported to reveal altered expression levels under diabetic conditions in other studies (26,27). Besides these six miRNAs, the present study identified 13 additional novel miRNAs whose expression levels were changed in diabetic GK rats, which may be future research targets for T2DM pathogenesis.

Through gene expression profiling, a total of 670 differentially-expressed genes were identified in the present study. A GO analysis indicated that the ‘immune response’ was the most significantly enriched item and the ‘ECM-receptor interaction’ was identified as the most significantly enriched pathway. A gene set for the ‘immune response’ includes chemokine (C-C motif) ligand 2 (*Ccl2*), *Ccl7*, *Ccl11*, *Cxcl1*, interleukin 1B (*Il1B*), *Il6* and interferon regulatory binding factor 8, indicating that inflammation may be a significant aspect in the dysfunction of β-cells in spontaneous T2DM GK rats. It is known that a T-cell-mediated autoimmune response against β cells is a primary pathogenic mechanism in type 1 diabetes (28). Additionally, growing evidence has indicated the causative link between tissue inflammation and the onset of insulin resistance (29). However, the effect of inflammation on β cell function in T2D was only reported recently (30–32) and the present study provided additional evidence that inflammatory processes are involved in β-cell dysfunction. The gene set for the ‘ECM-receptor interaction’ includes *Col2a1*, *Col27a1*, integrin, beta 6, *Cd36*, neuronal cell adhesion molecule and fibronectin 1. This indicated that under diabetic conditions, islets also exhibit dysfunction in pathways correlated to cell adhesion, cell migration and ECM accumulation, pathological characteristics which are common to diabetic nephropathy and diabetic retinopathy (33,34).

In addition to the deregulation of numerous miRNAs and mRNAs in T2DM tissues, the present study also identified potential miRNA-mRNA regulatory correlations that could have significant roles in the pathogenesis of T2DM. Several miRNAs, including miR-485, were connected with a number of putative target genes and certain genes, including *Pea15a*, *Gucy1a3*, *Pdzk1ip1*, *Ctgf*, *Itpkc* and *Hspa5* were found to be regulated by several miRNAs. These miRNAs and mRNA genes can be regarded as nodes of the whole regulatory network and are indicated to have pivotal functions. In this regard, *Pea15a* has been reported to control glucose transport and is overexpressed in fibroblasts, as well as in skeletal muscle and adipose tissues in type 2 diabetes (35). *Ctgf* has also been implicated to be associated with kidney complications that may occur in type 2 diabetes (36,37). Furthermore, *Hspa5* was found increased in the pancreas of type 2 diabetes patients, indicating increased endoplasmic reticulum stress during this disease (38), and in a microarray-based gene expression study, *Itpkc* was found to be insulin-regulated (39). These various studies are supportive of several of the findings of the present study. Furthermore, it was attempted to evaluate the strength of identified miRNA-mRNA correlations and it was proposed that miRNA-mRNA pairs, including let-7f/*Col2a1* may also have significant roles in T2DM.

Finally, through GO and pathway analysis of putative targets of individual miRNA, it was identified that certain miRNAs, including let-7f, regulated target genes associated with drug metabolism; certain miRNAs, including miR-150, regulated target genes associated with the response to glucose stimulation; numerous miRNAs, including miR-145, regulated genes associated with cell proliferation and apoptosis; and numerous other miRNAs, including miR-194, regulated genes associated with the inflammatory response. This observation indicated possible regulatory functions of each miRNA as well as the association of specific target genes with regulated cellular pathways.

In conclusion, mRNA and miRNA microarray analyses of the pancreas islet of GK rats have been performed and miRNA-mRNA regulatory networks in T2DM GK rats have been constructed. This analysis revealed possible miRNA-mRNA regulatory correlations between numerous miRNAs and their potential target genes. Further functional studies are required in order to confirm these proposed miRNA-mRNA regulatory correlations and to quantify their potential biological significance.

## Figures and Tables

**Table I:** Nineteen differentially-expressed miRNAs between GK and Wistar rats.

Gene expression	miRNA	Fold change
Upregulated	rno-miR-214	12.7232
	rno-miR-199a-5p	7.2678
	rno-miR-150	5.8137
	rno-miR-199a-3p	4.3030
	rno-miR-351	3.5565
	rno-miR-145	2.7425
	rno-miR-764	2.6075
	rno-miR-497	2.3444
	rno-miR-92b	2.2852
Downregulated	rno-miR-7a	0.2137
	rno-miR-325-5p	0.2871
	rno-miR-485	0.3500
	rno-miR-708	0.3693
	rno-miR-344-3p	0.3794
	rno-let-7f	0.4109
	rno-miR-26b	0.4590
	rno-miR-129	0.4715
	rno-miR-29c	0.4907
	rno-let-7a	0.4970

GK, Goto-Kakizaki; miRNA, microRNA.

**Table II:** Number of target genes for top 10 nodal miRNAs and the number of regulatory miRNAs for top 10 nodal genes.

microRNA	Number of targets	Gene symbol	Number of microRNA
rno-miR-485	32	*Pea15a*	4
rno-miR-129	29	*Gucy1a3*	4
rno-miR-7a	23	*Pdzk1ip1*	4
rno-miR-214	19	*Ctgf*	4
rno-miR-145	18	*Itpkc*	4
rno-let-7a	17	*Hspa5*	4
rno-miR-29c	17	*Snx27*	3
rno-miR-92b	17	*Nid67*	3
rno-let-7f	16	*Atp6v1b2*	3
rno-miR-26b	16	*Sv2a*	3
		LOC501110	3
		*Ist1*	3
		*Pde4b*	3
		*Ccng1*	3
		*Sparc*	3
		*Eif4b*	3
		*Nras*	3
		*Acadl*	3
		*Sult1a1*	3
		*Txnip*	3

miRNA, microRNA; *Pea15a*, phosphoprotein enriched in astrocytes 15; *Gucy1a3*, guanylate cyclase 1, soluble, alpha 3; *Pdzk1ip1*, PDZK1 interacting protein 1; *Ctgf*, connective tissue growth factor; *Itpkc*, inositol 1,4,5-triphosphate 3-kinase C; *Hspa5*, heat shock protein 5; *Snx27*, sorting nexin 27; *Nid67*, nidogen 67; *Atp6v1b2*, adenosine triphosphatase, H^+^ transporting, lysosomal 56/58kDa, V1 subunit B2; *Sv2a*, synaptic vesicle glycoprotein 2A; *Ist1*, increased sodium tolerance 1; *Pde4b*, cyclic adenosine monophosphate-specific 3′,5′-cyclic phosphodiesterase 4B; *Ccng1*, cyclin G1; *Sparc*, secreted protein, acidic, cysteine-rich (osteonectin); *Eif4b*, eukaryotic translation initiation factor 4B; *Nras*, neuroblastoma RAS viral oncogene homolog; *Acadl*, acyl-CoA dehydrogenase, long chain; *Sult1a1*, sulfotransferase family, cytosolic, 1A, phenol-preferring, member 1; *Txnip*, thioredoxin-interacting protein.

**Table III:** Top 10 strongest miRNA-mRNA regulatory correlations.

miRNA	Gene symbol	FC	FC of gene	Distance between changes
rno-let-7f	*Col2a1*	0.4109	48.2899	50.72358
rno-let-7a	*Col2a1*	0.4970	48.2899	50.30197
rno-miR-485	*Tspan1*	0.3500	43.6812	46.53834
rno-miR-7a	*Col1a2*	0.2137	33.6643	38.34376
rno-miR-129	*Lum*	0.4715	25.2511	27.37199
rno-let-7f	*Cyp2c22*	0.4109	24.1817	26.61538
rno-miR-29c	*Pcolce*	0.4907	24.575	26.61291
rno-miR-29c	*Lrrc17*	0.4907	24.4755	26.51341
rno-let-7a	*Cyp2c22*	0.4970	24.1817	26.19377
rno-miR-7a	*Ctgf*	0.2137	20.6531	25.33256

miRNA, microRNA; FC, fold change; *Col2a1*, collagen, type II, alpha 1; *Tspan1*, tetraspanin 1; *Lum*, lumican; Cyp, cytochrome P; *Pcolce*, procollagen C-endopeptidase enhancer; Lrrc, leucine rich repeat containing; *Ctgf*, connective tissue growth factor.

## Abbreviations

T2DM: type 2 diabetes
miRNA: microRNA
GK: Goto-Kakizaki
GO: gene ontology
MAS: molecule annotation system
ECM: extracellular matrix
FBS: fetal bovine serum
HBSS: Hank’s balanced salt solution
FDR: false discovery rate
FC: fold change
Pea15a: phosphoprotein enriched in astrocytes 15
Gucy1a3: guanylate cyclase 1, soluble, alpha 3
Pdzk1ip1: PDZK1-interacting protein 1
Ctgf: connective tissue growth factor
Itpkc: inositol 1,4,5-triphosphate 3-kinase C
Hspa5: heat shock protein 5
